# Quality assurance methodology for Varian RapidArc treatment plans

**DOI:** 10.1120/jacmp.v11i4.3164

**Published:** 2010-09-01

**Authors:** Ileana Iftimia, Eileen T. Cirino, Li Xiong, Herbert W. Mower

**Affiliations:** ^1^ Radiation Oncology Department Lahey Clinic Burlington MA; ^2^ Tufts University School of Medicine Boston MA USA

**Keywords:** patient‐specific RapidArc QA, MapCHECK/MapPHAN 5 2D diode array, Exradin A16 ion chamber, QUASAR phantom, RadCalc MU calculation

## Abstract

With the commercial introduction of the Varian RapidArc, a new modality for treatment planning and delivery, the need has arisen for consistent and efficient techniques for performing patient‐specific quality assurance (QA) tests. In this paper we present our methodology for a RapidArc treatment plan QA procedure. For our measurements we used a 2D diode array (MapCHECK) embedded at 5 cm water equivalent depth in MapPHAN 5 phantom and an Exradin A16 ion chamber placed in six different positions in a cylindrical homogeneous phantom (QUASAR). We also checked the MUs for the RapidArc plans by using independent software (RadCalc). The agreement between Eclipse calculations and MapCHECK/MapPHAN 5 measurements was evaluated using both absolute distance‐to‐agreement (DTA) and gamma index with 10% dose threshold (TH), 3% dose difference (DD), and 3 mm DTA. The average agreement was 94.4% for the DTA approach and 96.3% for the gamma index approach. In high‐dose areas, the discrepancy between calculations and ion chamber measurements using the QUASAR phantom was within 4.5% for prostate cases. For the RadCalc calculations, we used the average SSD along the arc; however, for some patients the agreement for the MUs obtained with RadCalc versus Eclipse was inadequate (discrepancy>5%). In these cases, the plan was divided into partial arc plans so that RadCalc could perform a better estimation of the MUs. The discrepancy was further reduced to within ~4% using this approach. Regardless of the variation in prescribed dose and location of the treated areas, we obtained very good results for all patients studied in this paper.

PACS number: 87.55.Qr

## I. INTRODUCTION

RapidArc (Varian Medical Systems, Palo Alto, CA) is a new treatment planning and delivery approach based on a volumetric intensity‐modulated single arc technique originally investigated by Otto.^(^ ^1^ ^)^ Later, Yu et al.^(^ ^2^ ^,^ ^3^ ^)^ and Duthoy et al.^(^ ^4^ ^,^ ^5^ ^)^ analyzed the benefits of using multiple coplanar or noncoplanar intensity‐modulated arcs for complex treatments, demonstrating that this new approach offers equivalent or superior target coverage, and highly improves sparing for critical structures compared to conventional conformal treatments.

RapidArc aims to improve the sparing for critical organs and healthy tissue compared to other intensity‐modulated radiation therapy (IMRT) solutions, to maintain at least the same degree for target coverage, and to significantly reduce the treatment time. Recently, the Varian RapidArc has become available for the treatment planning and delivery of arc‐dynamic IMRT. The arc optimization algorithm, PRO (Progressive Resolution Optimizer), optimizes leaf position, dose rate and gantry speed. The optimization is performed using progressive sampling in five resolution levels. In RapidArc version 8.5, the optimization begins with 10 control points, gradually increasing them (up to a maximum of 177). Both the treatment planning and linac systems must incorporate special capabilities such as variable dose rate, variable gantry speed and dynamic MLC movement in order to make a RapidArc plan deliverable.

With the commercial introduction of this new modality for treatment planning and delivery, the need has arisen for reliable and efficient techniques for performing patient‐specific RapidArc QA tests. RapidArc users have tried many methods for patient QA, using a variety of QA devices such as: a 2D ion chamber array, a 2D diode array, film alone or combinations of film‐diodes/film‐ion chambers in cylindrical phantoms, Bang gel, electronic portal imaging device, DynaLog files analysis, etc. However, publication of such work is scarce and does not describe in detail the QA methodology.^(^ ^6^ ^–^ ^11^ ^)^ Recently, 3D QA devices such as ArcCHECK (Sun Nuclear Corp., Melbourne, FL) and Delta4 (ScandiDos, Ashland, VA) were designed and tested for arc therapy QA.^(^ ^12^ ^,^ ^13^ ^)^

We implemented the Varian RapidArc in our clinic in early 2009. We performed the commissioning tests following the procedure developed by Ling et al.^(^ ^14^ ^)^ to check the hardware functionality (gantry speed and dose rate modulation). At that time, there was very little information regarding the patient‐specific QA; therefore, we decided to develop our own methodology. In this paper we present the methods and materials used in developing our QA procedure, along with the results we obtained. The information presented in this paper may help other RapidArc users to more easily start a QA program in their own clinics. In addition, this methodology may be used for patient‐specific QA for other rotational therapy approaches.

## II. MATERIALS AND METHODS

In our clinic for routine IMRT QA we measure each field individually in beam's eye view (BEV) (i.e., the gantry is set to 0° for each beam – IEC scale), using MapCHECK version 4.1.1 (Sun Nuclear Corp.), with added buildup for comparison with planar doses at a depth of 5 cm water equivalent. All IMRT plans are performed using Philips Pinnacle^3^ treatment planning software (Phillips Medical Systems, Bothell, WA), without considering couch attenuation for patient planning or QA.

For a RapidArc plan, we wanted to deliver the whole arc as planned. There were some details that we did not take into account for the IMRT QA which should now be considered, such as heterogeneity and rotational geometry. All RapidArc treatment plans described in this paper were generated using the Varian Eclipse treatment planning software version 8.5 (Varian Medical Systems, Palo Alto, CA). The measurements were performed on our Varian Trilogy machine (Serial Number 3560), with the 4D Treatment Console version 8, build 8.3.0.12.

When delivering arcs with the length greater than $\sim 340^{\circ }‐350^{\circ }$, the posterior portion of the beam goes through the couch and often through some part of the rails. For all RapidArc plans (QA calculations and measurements, and patient treatments), we limited the arc length to 350° and set the rails to the IN position. We did this to reduce the beam attenuation effect through the couch and rails.

The first step we took in establishing a QA methodology for a RapidArc plan was to validate the couch model in Eclipse by measuring the couch and rails attenuation for our Varian Trilogy machine's Exact couch with flat panel. The measurements were performed for the 6 MV photon energy with a cylindrical Exradin A12 ion chamber (Standard Imaging, Middleton, WI) in a solid water phantom, with 3 cm buildup in both anterior and posterior directions relative to the ion chamber plane. We found that the couch attenuation is ≈1.5%, while the couch and rails attenuation is ≈11.4% for beams going straight up from the posterior direction. These results are in agreement with data from other authors.^(^ ^15^ ^)^

The CT numbers for our Varian Exact couch support with flat panel and for the rails were obtained iteratively by doing calculations in Eclipse such that the attenuation introduced by these structures matched the measured values for our Trilogy couch. The calculations were performed in Eclipse using a water phantom exposed with a 6 MV posterior photon beam. This setup corresponds to the couch and rails attenuation measurements described above. We found that the CT number for our 2 cm thick solid couch could be set to ‐1000 for couch interior and ‐700 for couch surface, and the CT number for the 8 cm thick rails could be chosen as ‐150 to provide the proper attenuation. These results are in good agreement with data obtained by other RapidArc users.^(^ ^16^ ^)^

The maximum gantry rotation for a RapidArc plan is 358° (e.g., from 178° to 180° for a CCW rotation using IEC convention). For all patient plans presented here we were able to avoid the rails (placed in the IN position) by using a maximum gantry rotation range of 350° (from 175° to 185° for a CCW rotation).

**Figure 1:**
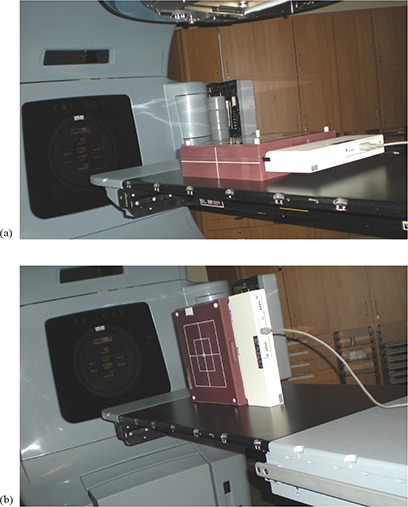
Photos with the MapCHECK/MapPHAN 5 device placed: a) horizontally on the couch to measure the dose distribution in the coronal plane; b) vertically to measure the dose distribution in the sagittal plane.

Given our experience with MapCHECK, we decided to add the MapPHAN 5 phantom (Sun Nuclear Corp., Melbourne, FL) for the RapidArc QA. The MapCHECK device, a two‐dimensional diode array with 445 detectors, was embedded at 5 cm water equivalent depth in MapPHAN 5 phantom and placed on the couch at isocenter. The MapCHECK/MapPHAN 5 device was placed horizontally on the couch to measure the dose distribution in the coronal plane, and vertically to measure the dose distribution in the sagittal plane (see photos in Figs. 1(a) and (b)).

Before we started using the MapCHECK/MapPHAN 5 phantom for the RapidArc QA, we performed the absolute calibration for this QA device. The calibration was performed right after the output was checked with the monthly QA setup for the 6 MV photon beam. The calibration procedure is the same as the one used for IMRT QA, except the MapPHAN 5 phantom is used as buildup.

The next step was to image the phantom in order to perform a RapidArc plan using these images and compare the planar doses with the MapCHECK/MapPHAN 5 measurements. Using our Philips AcQSIM Wide Bore CT we scanned the MapCHECK/MapPHAN 5 phantom, placing BBs on the crosshairs. The scans were performed in two different geometries: a) phantom placed horizontally, and b) phantom placed vertically on the CT couch, with the detectors facing toward the right side of the couch.

Two QA phantoms (coronal and sagittal) were created in the Varian Eclipse treatment planning software. The corresponding MapCHECK/MapPHAN 5 CT images were imported into Eclipse. Couch structures (couch and rails in the IN position) were attached to these images in Eclipse. The CT numbers used for the couch structures are given above.

All the RapidArc treatment plans were performed with 6 MV photon energy, using two equally‐weighted arcs (first arc CW and second arc CCW), with a typical gantry range of 350° (from 185° to 175° for the CW rotation and from 175° to 185° for the CCW rotation), using 177 control points for each arc. The maximum dose rate was set to 600 MU/min. The two‐arc approach was chosen in order to improve the dose homogeneity inside of the planning target volume (PTV). Prior to performing the optimization, we preset the collimator angle to 45° for the CW arc and to 315° for the CCW arc to minimize the tongue‐and‐groove effect. Because Eclipse 8.5 does not allow simultaneous optimization for multiple arcs, the two arcs were placed in separate plans and the arc optimization was performed independently until an adequate plan was obtained (i.e., meeting all established objectives). Even though the prescribed dose and the tolerance doses for critical structures were equally divided between the two arcs, the second arc used modified criteria to meet the final objectives. The two final plans were summed together and the dose‐volume histogram (DVH) for tumor and critical structures was analyzed to check if the criteria were met. The final calculations were performed without heterogeneity corrections using the Analytical Anisotropic Algorithm (Eclipse AAA 8223), with a voxel size of $2.5\times 2.5\times 2.5\;{\text{mm}}^{3}$. For localization purposes, the patients were imaged prior to each treatment by using either kV–kV or cone beam CT imaging tools. We did not account for any organ motion in the planning, treatment or QA tests. The couch structures (couch and rails) were not considered for the patients' plans, but they were avoided at least partially as mentioned above by leaving a posterior gap in the gantry rotation and by moving the rails IN for the treatment.

For our preliminary RapidArc QA tests we performed homogeneous calculations in phantom without considering couch structures, and heterogeneous calculations with couch structures included. For the work presented in this study, we decided to minimize the errors caused by any mismatch between the measurement and the calculation setup. Since all in‐phantom measurements described below were performed using the Varian Exact couch with the rails IN and the phantoms are heterogeneous to some extent, we decided to perform all calculations for the QA plans considering the couch structures and heterogeneity corrections. Even though we did not include the couch structures for the patient plans, we decided to include them for all QA plans because the thickness of the phantom is much smaller than the patient anterior‐posterior separation and a 10° posterior gap in gantry rotation may not be sufficient to avoid the rails for in‐phantom measurements.

Verification plans were created from the RapidArc treatment plans (CW and CCW) using the MapCHECK/MapPHAN 5 phantom's CT datasets (coronal and sagittal) as the reference images upon which the dose was calculated. Using the BBs, a beam isocenter was placed in the center of the detector plane. The calculations were performed in the phantom using the AAA algorithm, with preset MUs, MLC shapes, gantry speed and modulated dose rate from the patient plan. The planar doses were exported to the MapCHECK software in absolute mode, with a matrix size of $20\times 20{\text{cm}}^{2}$ and 202 points in both x and y directions (corresponding to a resolution of 0.1 cm). For the horizontal phantom, the planar doses were exported from the coronal view, while for the vertical phantom the planar doses were exported from the sagittal view.

The measurements for the RapidArc plans were performed for both arcs (CW and CCW), in both geometries (coronal – horizontal phantom; sagittal – vertical phantom), with the rails moved to the IN position. The MapCHECK/MapPHAN 5 calibration was checked by running the machine in isocentric setup (SAD 100 cm), for 6 MV, 108 MUs, gantry 0°, field size $10\times 10{\text{cm}}^{2}$, before each RapidArc plan QA. For this setup, the output should be 100 cGy. We decided that a ±2% output variation is acceptable, since this is the standard variation range recommended in the current Task Group Reports (#40 and #142).^(^ ^17^ ^,^ ^18^ ^)^ for the monthly check of the machine output. The measurements and calculations were compared using the MapCHECK software. The comparison was carried out as absolute dose. The agreement between calculation and measurement was evaluated using DTA and gamma index, with 3%/3 mm criteria. The DTA and gamma index were evaluated for detector positions receiving a dose larger than a threshold value set to 10% of the maximum dose (TH=10). In our clinic we use the DTA approach for the IMRT QA. We decided to also use the DTA approach for the RapidArc plans QA. The results using the gamma index approach are presented here only for comparison. These quantitative analysis techniques have been described in detail in the literature.^(^ ^19^ ^,^ ^20^ ^)^

**Figure 2:**
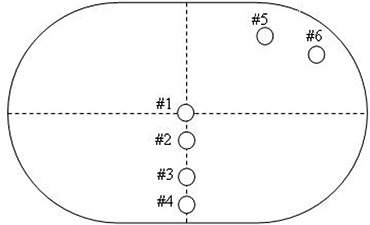
Schematic diagram of the QUASAR phantom. Points 1–6 were used for measurements and calculations. An Exradin A16 ion chamber was placed in turn in one of the six locations, and then the QA plan was run (CW and CCW). The reading was converted to dose and compared with the Eclipse calculations.

We decided to check the dose for some RapidArc plans as well using an ion chamber. We used a QUASAR (Quality Assurance System for Advanced Radiotherapy) multi‐purpose body phantom (Modus Medical Devices, Inc., London, ON, Canada), without any heterogeneous inserts. This decision was taken because of the cylindrical geometry of this phantom and also for the reason that we can perform measurements at various locations. An Exradin A16 cylindrical ion chamber placed in an appropriate holder was inserted in the middle of the QUASAR phantom. BBs were placed on crosshairs, and then the phantom was CT‐scanned with a 3 mm slice thickness, using our Philips AcQSIM Wide Bore scanner. The images were exported to Eclipse and linked to a new QA phantom (Quasar) created for this purpose. As in our procedure for the MapPHAN phantom, we attached the couch structures to these CT images, using the appropriate CT numbers to match the measured attenuation for the couch components of our Trilogy machine. A verification plan was created from the RapidArc treatment plan using the QUASAR phantom's CT dataset as the reference image used for dose calculation. The computation was performed using the AAA algorithm with a 2 mm calculation grid, and with heterogeneity corrections included (to take into account the ion chamber size, materials and various positions in the phantom). The measurements were performed by running the RapidArc QA plan on the QUASAR phantom with the rails IN. The Exradin A16 ion chamber was placed in six different locations (see Fig. 2) and both QA plans (for the CW and CCW arcs) were run one time in each of the six locations. The six points were distributed in the QUASAR phantom such that some were located in the high‐dose area, while others were located in the low‐dose area. Prior to these measurements, the Exradin A16 ion chamber was cross‐calibrated against a Holt Memorial parallel plate ion chamber (MPPK 154, CNMC Company, Inc., Nashville, TN), currently used for our monthly QA measurements for the Trilogy machine. The calibration was performed in polystyrene, at 5 cm water equivalent depth (SAD 100 cm), and $10\times 10{\text{cm}}^{2}$ field size. A reading‐to‐dose conversion factor was obtained for the Exradin A16 chamber and used to convert the reading to dose for our measurements.

The calculated dose for each of the six points was obtained from the Eclipse software by using the “point dose” feature. The point dose was read at the chamber position on the central axial CT slice. We then compared the measurements with the calculations.

We also performed a second check for all our RapidArc plans using independent software for MU calculations (RadCalc, version 5.2, LifeLine Software, Inc., Tyler, TX). The SSD listed in Eclipse is the value for the arc starting point. For a more realistic computation in RadCalc and for the purpose of improving the agreement with Eclipse, we took the average SSD (PSSD parameter) from the Eclipse full report and exported this value to RadCalc. Depending on patient anatomy, the PSSD can differ from the arc starting point SSD by a few centimeters. Using the arc starting point SSD can increase the discrepancy between RadCalc and Eclipse MU calculation from less than 5% to ~10%–12%. If the discrepancy was greater than 5% for either CW and/or CCW arcs, we used the following approach: we divided that arc into nine partial arcs, each partial arc but the last having 20 control points (i.e., 1–20,21–40,‥,161–177). Each partial plan was computed in Eclipse with preset MUs for that portion of the arc, approved and exported to RadCalc with the average SSD for that partial arc. The higher the number of partial arc plans, the better the agreement. We decided to use nine partial arc plans because the SSD for this gantry range $\left(\sim 40^{\circ }/\text{partial}\;\text{arc}\right)$ is reasonably approximated in RadCalc.

Prior to performing our first patient‐specific QA following the approach described in this paper, we tested and fine‐tuned our methodology by doing an end‐to‐end run for a “test patient.” Finally, to further improve our efficiency in performing the RapidArc plan QA, we wrote user‐friendly, step‐by‐step procedures for all parts (dose calculations, measurements, comparisons, etc.) considered in this methodology.

### III. RESULTS & DISCUSSION

In this study, we included eleven RapidArc patients: four prostate, one prostate cone down (first course being an IMRT for prostate and nodes), two prostate bed, one trachea, and three GYN. For the GYN patients, the prescribed dose was 2500 cGy, with 500 cGy per fraction while, for all other patients, the dose per fraction was 180 cGy, and the number of treatment fractions varied (41 for prostate, 16 for prostate cone down, 37 for prostate bed, and 36 for trachea). The total MU per treatment fraction for these plans was in the range of 1400–1800 for the GYN cases, and about 400–750 for other cases, depending on the plan complexity. The percent isodose line used for normalization was ≈80%–90% for the GYN cases, and 94%–97% for the others.

We performed ten RapidArc plans using two arcs (first CW, second CCW), with a gantry range of 350° (from 185° to 175° for the CW rotation and from 175° to 185° for the CCW rotation), using 177 control points for each arc. The patient with tracheal carcinoma was treated previously for a head and neck cancer and the cord dose was already close to the tolerance. To avoid the cord, we reduced the gantry rotation range slightly more for this case (i.e., we used a gantry range of 340°, from 190° to170° for the CW rotation and from 170° to 190° for the CCW rotation). We set the collimator angle to 45° for the CW arc and to 315° for the CCW arc. The arc optimization was performed independently for the two arcs and then the two plans were summed. The DVH was analyzed for tumor and critical structures to check if all objectives were met. The final calculations were performed using the AAA algorithm without heterogeneity corrections and without couch structures considered.

For all patients, we performed MapCHECK/MapPHAN 5 measurements and RadCalc calculations as described in the section above. Only three patients were selected for the QUASAR measurements (one prostate, one prostate bed, and one GYN patient).

To quantify the effect of heterogeneity corrections, in our preliminary RapidArc QA tests we performed homogeneous calculations in phantom without considering couch structures, and heterogeneous calculations with couch structures included. When homogeneous calculations were compared with heterogeneous calculations, the percent agreement (i.e., the percentage of diodes meeting the acceptance criteria ‐ absolute DTA approach, TH=10,3%/3mm) between MapCHECK/MapPHAN 5 measurements and Eclipse calculations decreased with up to ~28% for the coronal plane, and up to ~13% for the sagittal plane. When absolute gamma index approach with TH=10,3%/3mm criteria was used, the decrease in percent agreement was slightly less. The percent agreement for the CAX diode also decreased with up to ~5% when homogeneous calculations were used. As we expected, the best agreement between the measurements and calculations was obtained when we used heterogeneity corrections and couch structures, because this calculation setting matches the experimental one.

**Figure 3:**
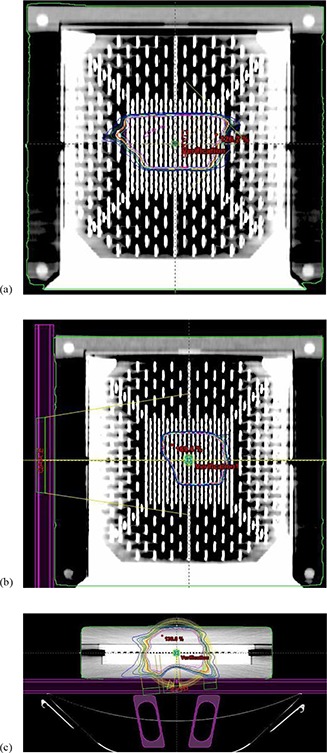
The calculated dose for a prostate patient: a) in the coronal plane using the horizontal phantom; b) in the sagittal plane using the vertical phantom; c) the axial plane from the horizontal phantom showing the couch and rails structures considered for the dose computation.

We performed MapCHECK/MapPHAN 5 measurements for both arcs (CW and CCW), with the phantom horizontal and vertical, as mentioned in the description of the QA method. We computed the planar doses in the coronal and sagittal planes and compared them with the measurements using the MapCHECK software. Figures 3(a) and (b) show the calculated dose for a prostate patient in the coronal plane using the horizontal phantom, and in the sagittal plane using the vertical phantom, respectively. The axial plane from the horizontal phantom displayed in Fig. 3(c) shows the couch and rails structures included for the in‐phantom dose computation. The agreement between the calculated and the measured dose was evaluated using both absolute DTA and gamma index, for TH=10,3%/3mm criteria (see Table 1). The range for the percent agreement was 89.1%–99.1% (average agreement: 94.4%) for the DTA approach and 90.4%–100% (average agreement: 96.3%) when the gamma index approach was used. The percent discrepancy for the CAX diode is also shown in Table 1.

**Table 1:** MapCHECK – Eclipse comparison.

	*CW Coronal*	*CW Sagittal*	*CCW Coronal*	*CCW Sagittal*
	*Average % agreem.*	*Average % agreem.*	*Average % agreem.*	*Average % agreem.*
DTA Gamma	93.5% 95.4%	96.5% 97.9%	94.0% 95.7%	93.7% 96.2%
	*CAX Average % discrepancy*	*CAX Average % discrepancy*	*CAX Average % discrepancy*	*CAX Average % discrepancy*
CAX $\left[\text{meas.‐calc.}]/{\text{calc}}^{*}100(\%\right)$	‐3.5%	‐3.5%	‐2.2%	‐2.8%

Percentage of diodes receiving a dose greater than 10% of the maximum dose that met the acceptance criteria (3%/3 mm) for DTA and gamma index approaches, and percent discrepancy of the CAX diode.

**Figure 4:**
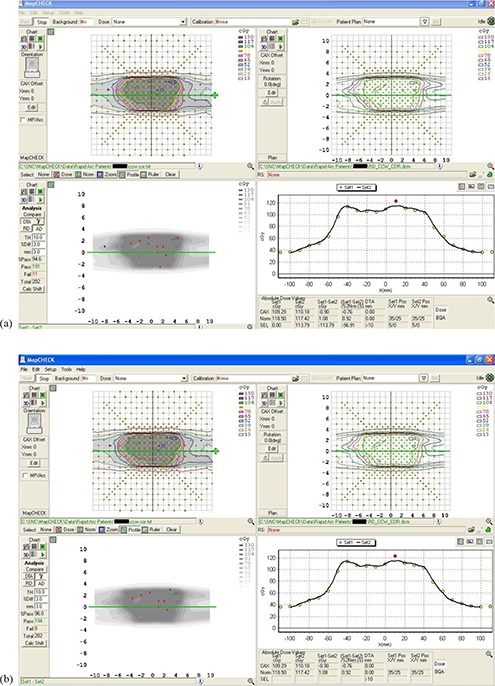
A coronal view of a CCW RapidArc plan (right side, upper panel), compared with the measurements (left side, upper panel) by using: a) the DTA approach; b) the gamma index approach. The image displayed in the left side of the lower panels shows the difference between the measurements and the calculations. In the right side of the lower panels there is a graph showing a horizontal profile. The solid line in the graph represents the calculation, while the dots correspond to the measured values.

**Figure 5:**
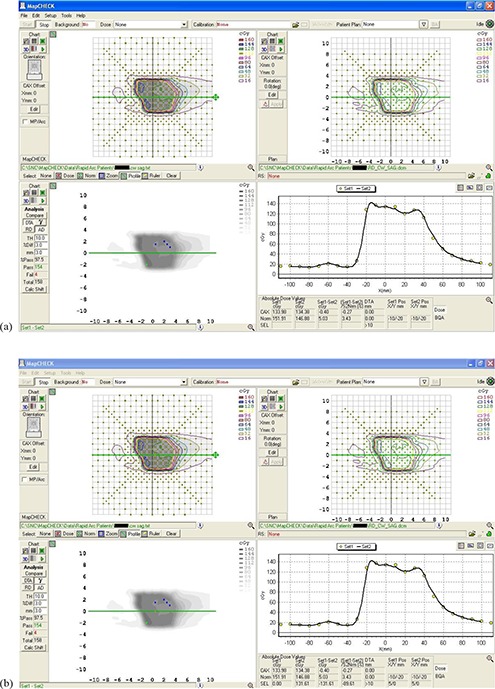
A sagittal view of a CW RapidArc plan (right side, upper panel), compared with the measurements (left side, upper panel) by using: a) the DTA approach; b) the gamma index approach. The image displayed in the left side of the lower panels shows the difference between the measurements and the calculations. In the right side of the lower panels there is a graph showing a horizontal profile. The solid line in the graph represents the calculation, while the dots correspond to the measured values.

As an example, Figs. 4(a) and (b) and 5(a) and (b) display screenshots from the MapCHECK software showing the results of delivery of a prostate plan. Figures 4(a) and (b) show the coronal view of a CCW RapidArc plan (right side, upper panel), compared with the measurements (left side, upper panel) by using a) the DTA approach and b) the gamma index approach. Similarly, Figs. 5(a) and (b) show the sagittal view of a CW RapidArc plan (right side, upper panel), compared with the measurements (left side, upper panel) by using a) the DTA approach and b) the gamma index approach. In the upper panels of Figs. 4 and 5, the color coding shows the absolute dose from measurements (left side) and calculations (right side). The image displayed in the left side of the lower panels shows the difference between the measurements and the calculations. (The blue and red dots are the “failing points” – i.e., the ones which do not pass the selected criteria of TH=10,3%/3mm). In the right side of the lower panels, there is a graph showing a horizontal profile. The solid line in the graph represents the calculation, while the dots correspond to the measured values. The agreement between measured and calculated dose is very good for this patient, only slightly better for the gamma approach than for the DTA approach.

**Figure 6:**
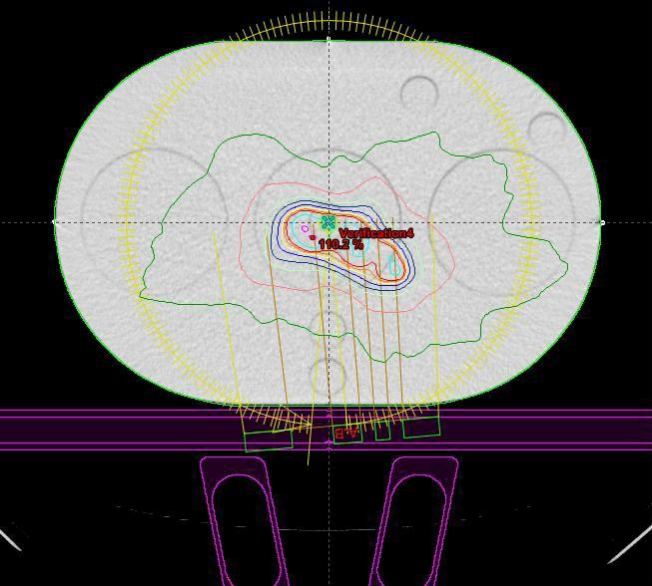
The axial view of a GYN verification plan computed using the QUASAR phantom's CT dataset. Couch and rails in the IN position are considered for dose calculation. The six locations for the ion chamber during measurements are displayed as small circles.

Figure 6 shows the axial view of a GYN verification plan using the QUASAR phantom's CT dataset for dose computation. As it can be seen in this Figure, couch and rails in the IN position were considered for dose calculation. The six locations for the ion chamber during measurements are displayed as small circles and correspond to the points drawn in the schematic diagram in Fig. 2. The agreement between the measurements performed using the QUASAR phantom and Eclipse calculations was good for the prostate patients (within 4.5% discrepancy), for both points 1 and 2 located in high‐dose area. For the GYN patient the percent discrepancy was within 3% for point 1 and within 8% for point 2. This can be explained by the high dose gradient in the region were point 2 was located for this QA plan (see Fig. 6). For the points located in low‐dose area (points 3 to 6), the discrepancy was less than ≈7.5% for the GYN patient and less than ≈11.5% for the other two patients. The difference between the measured and the calculated dose for the points 3 to 6 was smaller than 4–5 cGy. (The dose in low‐dose areas was about 60–70 cGy for the GYN patient and 20–40 cGy for the other two patients.) In addition, another small potential source of error may arise due to the fact that the heterogeneous plan was calculated with the ion chamber placed in the middle of the phantom, while for the measurements it was located in turn in one of the six points used for this QA test. Table 2 shows the comparison between QUASAR/A16 ion chamber measurements and Eclipse calculations. The measurements points are separated as shown in two categories. Points 1 and 2 were located in high‐dose area (dose >80% of prescribed dose), while the other points (3 to 6) were located in lower dose areas (dose <80% of prescribed dose). The standard deviation is better for points 1 and 2, as expected.

**Table 2:** QUASAR/A16 ion chamber measurements – Eclipse calculation comparison.

*Statistical Analysis*	% Discrepancy Points 1 & 2 Dose>80% *of Prescribed Dose*	*% Discrepancy Points 3, 4, 5, & 6* Dose<80% *of Prescribed Dose*
Average	1.16%	0.53%
Median	1.30%	‐0.10%
STDEV	4.36%	6.82%

The comparison between QUASAR/A16 ion chamber measurements and Eclipse calculations. The measurements points are separated in two categories. Points 1 and 2 were located in high‐dose area (dose>80% of prescribed dose), while the other points (3 to 6) were located in lower dose area (dose<80% of prescribed dose).

Although for the RadCalc calculations we used the average SSD along the arc (PSSD parameter from Eclipse full report), for some patients the agreement for the MUs obtained with RadCalc versus Eclipse was inadequate (discrepancy>5%; however, it still was not >9%). This happened for both arcs (CW and CCW) for two GYN patients, for which the right‐left separation was much greater than the anterior‐posterior separation, so the PSSD was not a good approximation for the SSD at various gantry angles along the arc. Even for some prostate patients the agreement was relatively poor (5%–7% discrepancy). If the discrepancy between RadCalc and Eclipse was greater than 5%, we divided the plan into nine partial arc plans and exported these partial arc plans to RadCalc, considering the average SSD for that portion of the arc. The agreement between the Eclipse MUs and the MUs summed over all nine partial arcs computed in RadCalc was much better (usually 1%–2% discrepancy; not more than about 4%). The results for this comparison are presented in Table 3. We noticed that the percent difference between RadCalc and Eclipse was always negative (i.e., MUs from RadCalc were smaller than MUs from Eclipse). That could indicate a systematic error in the RadCalc and/or Eclipse software used in this study.

**Table 3:** RadCalc – Eclipse comparison.

*No.*	*Site*	*% Discrepancy RadCalc CW*	*% Discrepancy RadCalc CCW*	*% Discrepancy RadCalc CW 9 Partial Arcs*	*% Discrepancy RadCalc CCW 9 Partial Arcs*
1.	GYN	‐8.0%	‐9.0%	‐2.1%	‐3.6%
2.	GYN	‐5.9%	‐5.4%	‐4.2%	‐4.2%
3.	Trachea	‐3.7%	‐4.3%	‐	‐
4.	Prostate	‐4.2%	‐5.1%	‐	‐0.7%
5.	Prostate	‐7.4%	‐2.6%	‐1.3%	‐
6.	Prostate	‐3.6%	‐3.5%	‐	‐
7.	Prostate	‐1.2%	‐1.4%	‐	‐
8.	Prostate Bed	‐3.0%	‐5.1%	‐	0%
9.	CD Prostate	‐4.3%	‐7.2%	‐	‐2.6%
10.	GYN	‐3.5%	‐3.7%	‐	‐
11.	Prostate Bed	‐4.0%	‐7.3%	‐	‐0.5%
	Average	‐4.44%	‐4.96%	‐3.19%	‐2.46%
Statistical	Median	‐4.00%	‐5.10%	‐3.60%	‐2.60%
Analysis	STDDEV	1.96%	2.22%	1.15%	1.56%
	Range	(‐)1.2%–(‐)8%	(‐)1.4%–(‐)9%	(‐)1.2%–(‐)4.2%	(‐)1.4%–(‐)4.3%

The comparison between the RadCalc MUs and the Eclipse MUs for the CW and CCW arcs for all patients studied. When the discrepancy was > 5% the arc was divided into nine partial arcs for a better MU estimation in RadCalc. The agreement was much improved (see last two columns).

## IV. CONCLUSIONS

We developed a RapidArc patient‐specific QA methodology by using for our measurements a 2D diode array (MapCHECK) embedded at 5 cm water equivalent depth in MapPHAN 5 phantom and an Exradin A16 ion chamber placed in six different positions in a cylindrical homogeneous phantom (QUASAR). The agreement between Eclipse calculations and MapCHECK/MapPHAN 5 measurements was evaluated using both absolute DTA and gamma index, with TH=10,3%/3mm criteria. The average agreement was 94.4% for the DTA approach and 96.3% for the gamma index approach. It has been suggested that a higher dose threshold (i.e., TH=20) would be more appropriate for such analysis.^(^ ^21^ ^)^ Our preliminary results showed that when the threshold dose was increased from 10% to 20% of maximum dose, the percent agreement decreased by a maximum of 2.5%. We will investigate this in more detail in our future work.

In high‐dose areas, the discrepancy between calculations and ion chamber measurements using QUASAR phantom was within 4.5% for selected prostate patients.

Also, we checked the MUs for the RapidArc plans by using independent software (RadCalc). When the discrepancy was >5%, the plan was divided into partial arc plans so that RadCalc could perform a better estimation of the MUs. The discrepancy was further reduced to within ~4% using this approach. Despite the variation in prescribed dose, anatomy and location of the treated areas, we obtained very good results for all patients studied in this paper.

In summary, for all RapidArc plans, we evaluated both the DTA and gamma index approach for MapCHECK/MapPHAN 5 measurements – planar doses comparison and determined to use the DTA approach as we do for our routine IMRT QA. Currently we perform MapCHECK/MapPHAN 5 measurements in both coronal and sagittal planes. We performed the QUASAR measurements for only three patients. We used the partial arc plan approach only if the discrepancy between RadCalc and Eclipse calculations was greater than 5%. We did the partial arc plans only for nine out of 22 beams. The time needed to perform all these QA tests, including planar dose calculation, was about three hours per patient.

In the future we expect to perform MapCHECK/MapPHAN 5 measurements only in one of the two planes (coronal or sagittal). As we gain experience, we may be able to determine based on the treatment site which is the more appropriate plane for measurements. This will reduce both QA measurements and planar doses computation time. After initial testing, we discontinued QUASAR measurements. Future RadCalc software will be able to import the body contour; therefore, the SSD will be correctly estimated for each point along the arc. The calculation accuracy should improve resulting in better agreement with Eclipse calculations. Recently we upgraded the Eclipse software to version 8.6, which allows simultaneous optimization for multiple arcs. This will further reduce the time needed for planning and QA calculations. We are optimistic that the time involvement will become comparable to routine IMRT QA.

Intensity‐modulated arc therapy is a new and rapidly evolving technique. Current detectors are already providing very promising results, and we anticipate that in the future, enhanced detectors better adapted to the complexity of this approach will yield even further advances.

## ACKNOWLEDGEMENTS

We would like to thank our colleague Muazzam Kazi for the help he gave us in performing part of the measurements for this paper.
